# Role of the Renin-Angiotensin System in Long COVID’s Cardiovascular Injuries

**DOI:** 10.3390/biomedicines11072004

**Published:** 2023-07-15

**Authors:** Elena Cojocaru, Cristian Cojocaru, Cristiana-Elena Vlad, Lucian Eva

**Affiliations:** 1 Morpho-Functional Sciences II Department, Faculty of Medicine, “Grigore T. Popa” University of Medicine and Pharmacy, 700115 Iasi, Romania; elena.cojocaruu@umfiasi.ro; 2 Medical III Department, Faculty of Medicine, “Grigore T. Popa” University of Medicine and Pharmacy, 700115 Iasi, Romania; 3 Medical II Department, Faculty of Medicine, “Grigore T. Popa” University of Medicine and Pharmacy, 700115 Iasi, Romania; cristiana-elena.vlad@umfiasi.ro; 4 “Dr. C. I. Parhon” Clinical Hospital, 700503 Iasi, Romania; 5 Faculty of Dental Medicine, “Apollonia” University of Iasi, 700511 Iasi, Romania; lucianeva74@yahoo.com; 6 “Prof. Dr. Nicolae Oblu” Clinic Emergency Hospital, 700309 Iasi, Romania

**Keywords:** renin-angiotensin system, ACE2, angiotensin II, signal transduction, cardiovascular system, long COVID

## Abstract

The renin-angiotensin system (RAS) is one of the biggest challenges of cardiovascular medicine. The significance of the RAS in the chronic progression of SARS-CoV-2 infection and its consequences is one of the topics that are currently being mostly discussed. SARS-CoV-2 undermines the balance between beneficial and harmful RAS pathways. The level of soluble ACE2 and membrane-bound ACE2 are both upregulated by the endocytosis of the SARS-CoV-2/ACE2 complex and the tumor necrosis factor (TNF)-α-converting enzyme (ADAM17)-induced cleavage. Through the link between RAS and the processes of proliferation, the processes of fibrous remodelling of the myocardium are initiated from the acute phase of the disease, continuing into the long COVID stage. In the long term, RAS dysfunction may cause an impairment of its beneficial effects leading to thromboembolic processes and a reduction in perfusion of target organs. The main aspects of ACE2—a key pathogenic role in COVID-19 as well as the mechanisms of RAS involvement in COVID cardiovascular injuries are studied. Therapeutic directions that can be currently anticipated in relation to the various pathogenic pathways of progression of cardiovascular damage in patients with longCOVID have also been outlined.

## 1. Introduction

The first cases of COVID-19, the disease caused by the novel coronavirus SARS-CoV-2, were reported in December 2019 in the city of Wuhan, Hubei Province, China. However, the initial cluster of cases was reported to the World Health Organization (WHO) on 31 December 2019. This marked the beginning of global recognition and response to the COVID-19 pandemic. Due to this, on 30 January 2020, the WHO declared a Public Health Emergency of International Concern and on 11 March 2020 classified the outbreak as a pandemic [1]. Over 767 million confirmed cases and more than 6.9 million deaths had been reported globally as of 4 June 2023 [2]. According to WHO, no matter the age or the severity of the first symptoms, the individuals exposed to SARS-CoV-2 are susceptible to the post-COVID-19 disease, also known as long COVID [3,4]. Informal patient reports of previously healthy people enduring persistent symptoms and not fully healing following infection with SARS-CoV-2 began to appear in April 2020, not long after the pandemic’s onset. These patients began to identify as “Long Haulers,” and thus the term “Long COVID” was invented [5].

The Department of Health and Human Services, in collaboration with the Centers for Disease Control and Prevention and other partners, issued the Long COVID definition: “Long COVID is broadly defined as signs, symptoms, and conditions that continue or develop after initial COVID-19 or SARS-CoV-2 infection. The signs, symptoms, and conditions are present four weeks or more after the initial phase of infection; may be multisystemic; and may present with a relapsing–remitting pattern and progression or worsening over time, with the possibility of severe and life-threatening events even months or years after infection. Long COVID is not one condition. It represents many potentially overlapping entities, likely with different biological causes and different sets of risk factors and outcomes” [6]. 

Based on an estimation of 10% of infected individuals and more than 651 million COVID-19 cases worldwide, at least 65 million people worldwide have long-term COVID [7]. Long COVID can be divided into two phases based on how long the symptoms last. Post-acute COVID refers to symptoms that last longer than 3 weeks and up to 12 weeks, whereas chronic COVID refers to symptoms that last longer than 12 weeks [8,9].

Age and sex gender are related to long-COVID progression. In fact, women and/or young people have a higher probability of developing long COVID than men, but the risk level levels out around the age of 60 [10]. Regardless of whether individuals were hospitalized, a study found that 45% of COVID-19 survivors had a variety of unresolved symptoms after less than four months [11]. 

There is disagreement among experts on the type of symptoms that COVID-19 may be responsible for, which further complicates issues. Another study revealed that 12.7% of COVID-19 patients in the general population will experience persistent somatic symptoms after COVID-19 by considering the symptoms that worsened and could be linked to COVID-19 and while correcting for seasonal variations and non-infectious health aspects of the pandemic on symptom dynamics [7]. According to a study based on 9764 participants, the 12 main symptoms that characterize the most of the long COVID disease are: loss of smell or taste, post-exertional malaise, chronic cough, brain fog, thirst, heart palpitations, chest pain, fatigue, dizziness, gastrointestinal symptoms, issues with sexual desire or capacity, and abnormal movements. The prevalence of long COVID across the various study groups ranged from 10 to 23% based on the 12 defining symptoms, depending on when they got the disease and whether they were already diagnosed with long COVID when they enrolled in the study. In addition, this study revealed that long COVID was more prevalent in those who were unvaccinated and those who experienced it more than once [4].

It has been observed that in patients with a history of cardiovascular disease, higher serum concentrations of ACE2 may be responsible for the increased severity of COVID-19 disease [12]. Furthermore, the use of ACE inhibitor therapies may increase the risk of SARS-CoV-2 infection. However, in patients who have recovered, Ang II levels normalized [13]. Patients who have required a prolonged hospital stay or have died have associated elevated serum troponin levels as an expression of ischemic and non-ischemic cardiac lesions [14].

In addition to hemodynamic actions, local RAS has multiple functions, including regulation of cell growth, differentiation, proliferation, apoptosis, reactive oxygen species generation, tissue inflammation and fibrosis, and hormone secretion. In this regard, local RAS has alternative pathways regulated by biologically active peptides (e.g., Ang IV, Ang A, alamandine, and angioprotectin), additional specific receptors (e.g., pro-renin receptor), and alternative pathways for Ang II generation (e.g., renin-independent mechanisms of Ang-peptide generation from Ang (1–12)) [15]. The precise mechanisms of myocardial injury in patients with COVID-19 are still unclear. It is also not known whether the myocardial injury is a direct effect of the virus or a response to systemic inflammation or both.

Among the factors of poor prognosis in patients with COVID-19, older age, presence of comorbidities, and smoking were cited. A study published by Cheng et al. found that older age is associated with reduced ACE2 and consequently reduced susceptibility to infection [16].

## 2. Methods

We have conducted a systematic review of the literature to provide updated evidence on the role of RAS during the long COVID-19. The following databases have been searched: Medline, Google Scholar, and WHO Global Research Database on COVID-19 from 1 January 2020 to 10 June 2023. We screened original articles and reviews in English, and the search terms included “long COVID”, “renin-angiotensin system”, “ACE2”, “cardiovascular effects”, “WHO long COVID”, “SARS-COV-2”, as well as “persistent symptoms”.

## 3. The Alteration of RAS Components—A Key Role in SARS-CoV-2 Infection

The RAS is composed of a series of regulatory peptides that play important roles in the body’s physiological functions, such as maintaining fluid and electrolyte balance, controlling blood pressure, controlling vascular permeability, and promoting tissue growth [17]. 

**Figure 1:**
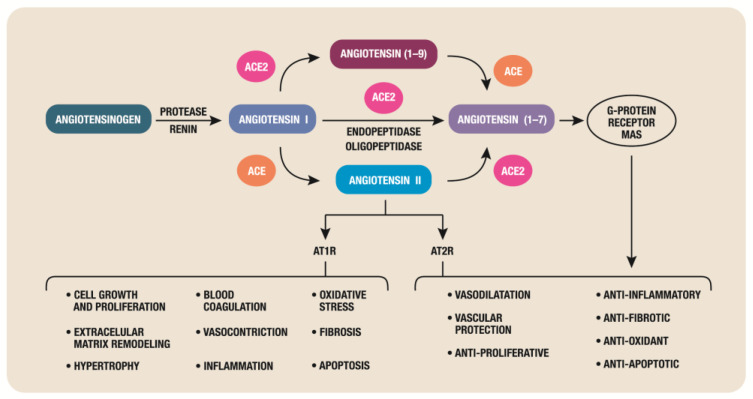
The two axes of the RAS.

RAS has two opposite axes [18] (Figure 1):

1. Harmful arm-ACE/Ang II/angiotensin II receptor type 1 (AT1R) pathway; because it separates Ang II from Ang I and interacts with the AT1R to cause vasoconstriction, inflammation, hypertrophy, blood coagulation, cell growth and proliferation, extracellular matrix remodelling, and stimulation of oxidative stress and fibrosis.
2. Protective arm-ACE2-Ang (1–7)-MasR receptor (MasR); Ang (1–7) interacts with MasR to promote vasodilation, anti-inflammatory effects, antifibrotic effects, anti-proliferative effects, and vascular protection and mediates endothelial nitric oxide synthase activation and suppress apoptosis, negatively regulating RAS. The Ang (1–7) is produced either by the cleavage of Ang II by ACE2 or through the metabolism of inactive Ang (1–9) (cleaved from Ang I by ACE2). The cardiac antihypertrophic actions of Ang II and Ang (1–7) are mediated through the activation of Ang type 2 receptors (AT2R) and MasR, respectively [19]. Since only AT1R is blocked, using an ARB (angiotensin receptor blockers) for the therapy of COVID-19 patients may have favorable outcomes because any formed Ang II may have anti-inflammatory effects through its interaction with AT2R or through conversion by residual ACE2 to Ang (1–7) acting through AT2R, MasR, and Mas-related G protein-coupled receptor D) [20]. Thus, by preventing or reversing Ang II-induced cardiac hypertrophy, AT1R inhibition or activation of AT2R and Mas receptors as well as decreased oxidative stress may have a cardioprotective role.

The balance between the two axes defines health status. Conditions including lung disease, hypertension, and heart failure have all been related to an imbalance between these two systems [21]. 

The key receptor at the SARS-CoV-2 entry, ACE2, is considerably reduced in COVID-19 infection, which inhibits the protective effect of the ACE2-Ang (1–7)-MasR arm. Dysregulation of the RAS, direct viral toxicity, endothelial cell destruction, and thrombo-inflammation, as well as dysregulation of the immune system, are among the mechanisms allowing multi-organ damage caused by SARS-CoV-2 infection. The involvement of these mechanisms in the pathophysiology of COVID-19 is currently not fully understood [18]. RAS dysregulation and ACE-2 mediated viral entry, as well as tissue damage, may be also secondary to sepsis, even though some of these mechanisms, such as systemic cytokine release and microcirculatory dysfunction, may be important factors to the pathogenesis of COVID-19.

Due to the involvement of ACE2, a key part of the RAS, this system has been linked to COVID-19. The RAS is stated to be involved in COVID-19 in various ways.

### 3.1. SARS-CoV-2 Cell Entrance

**Figure 2:**
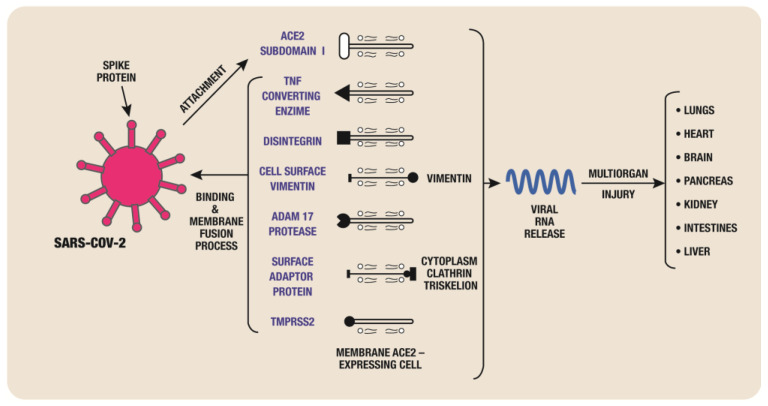
SARS-CoV-2 cell entrance.

The SARS-CoV-2 virus can enter human cells (Figure 2), particularly those in the respiratory tract, through the ACE2 receptor. The virus’s spike protein links to ACE2, allowing the virus to enter host cells more easily. An essential phase in the infection process is this interaction between the virus and ACE2.

A summary of the steps involved in the direct viral entrance is provided as follows:

3.1.1. The ACE2 receptor, which is widely expressed on the surface of a variety of human cells, including those in the respiratory tract, lungs, heart, and other organs, is recognized and bound to the spike protein on the surface of SARS-CoV-2 [18].

3.1.2. After initial attachment, the spike protein undergoes a process termed priming. Some transmembrane proteinases and proteins, including vimentin and clathrin, may be involved in the binding and membrane fusion processes. Examples include transmembrane protease serine 2 (TMPRSS2), disintegrin and ADAM17, and TNF-converting enzyme. For instance, both TMPRSS2 and ADAM17 have the ability to cleave ACE2 in order to increase viral uptake and ectodomain shedding, respectively [22,23,24,25]. However, additional proteases may also be involved in this process. The spike protein’s structural modifications brought on by priming enable it to merge with the host cell membrane.

3.1.3. Membrane Fusion: In order to merge with the host cell membrane, the primed spike protein undergoes a conformational shift. This fusion enables the virus to release its genetic material and enter the cytoplasm of the host cell.

3.1.4. Genetic Material Release: The viral genetic material, which takes the form of ribonucleic acid (RNA), is released once inside the host cell. The instructions for generating new viral components are encoded by the viral RNA.

3.1.5. Replication and Assembly: The mechanisms of the host cell use the viral RNA to create viral proteins and reproduce the viral genome. In the host cell, new virus particles are assembled.

3.1.6. Release of New Viral Particles: the newly formed viral particles are released from the infected host cell, either by cell lysis (cell apoptosis) or by budding from the host cell membrane. These released viral particles can continue to infect other cells and continue the cycle of infection.

Because of this, SARS-CoV-2 can infect different systems in the body, leading to a range of COVID-19-related symptoms and complications.

### 3.2. ACE2—A Key Pathogenic Role in COVID-19

The components of the RAS and, in particular, ACE2—the central element of this system—play a particular role in the pathological states of COVID-19 via the following mechanisms:

#### 3.2.1. ACE2 Downregulation

SARS-CoV-2 infection has been associated with the downregulation of ACE2 expression. The binding of the virus to ACE2 and its internalization during the infection process can lead to a decrease in the number of ACE2 receptors on the cell surface. This downregulation of ACE2 could have several implications, as ACE2 is involved in various physiological processes, including the regulation of blood pressure and the balance of the RAS.

#### 3.2.2. Imbalance in the RAS

ACE2 is responsible for converting Ang II to Ang (1–7), which has vasodilatory and anti-inflammatory effects. The downregulation of ACE2 during SARS-CoV-2 infection can result in an imbalance between Ang II and Ang (1–7) levels. This imbalance may contribute to increased levels of Ang II, which is known to promote vasoconstriction, inflammation, and fibrosis.

#### 3.2.3. Inflammatory Response and Lung Injury

Dysregulation of the RAS system, along with the release of pro-inflammatory cytokines and chemokines, can contribute to an excessive and uncontrolled inflammatory response in the lungs. This inflammatory response can lead to lung injury, acute respiratory distress syndrome (ARDS), and other complications associated with severe COVID-19.

#### 3.2.4. The Kallikrein-Kinin System (KKS)

KKS, the degradation pathways of which are regulated by ACE and ACE2, is a system linked to RAS. Through the cleavage of kininogens by kallikrein, the KKS increases in inflammation and causes an increase in bradykinin [26]. An increased risk of thrombotic events such as pulmonary embolism, myocardial infarction, and stroke is reported after COVID-19 infection [27]. Considering that the kallikrein–kinin system can activate the coagulation system through factor XII, therapeutical approaches that rebalance the RAS and the closely linked KKS may be able to reduce the hyperinflammatory and procoagulant state experienced by long-COVID patients [28]. Since blocking the AT1R restores RAS equilibrium as a result of a reduction in ADAM17, an increase in ACE2 subsequently, and a downregulation of the B1 bradykinin receptor, targeting RAS in long-COVID might be favourable. Studies have shown that AT1R antagonists/blockade (AT1RB) are beneficial for long-COVID patients because they prevent the ACE-dependent degradation of bradykinin [29,30]. Using computational modelling, it was recently discovered that AT1RB could be changed to have a stronger affinity for the ACE2-receptor, competing with or scavenging for the spike protein of the SARS-CoV-2 [29]. Targeting RAS has not been found to benefit outcomes for adult patients hospitalized for acute COVID-19, according to three recent studies. Based on an assumption that overactivation of the RAS pathway would result in worse COVID-19 results, the REMAP-CAP investigators have studied the use of an ACE inhibitor or an ARB against no RAS inhibition [31]. In two trials, TXA-127 and TRV-027, respectively, which have both been evaluated versus placebo, researchers from the ACTIV-4 Host Tissue platform got a novel approach to correct imbalances in Ang (1–7) and Ang II caused by RAS dysregulation [32,33]. However, it should be noted that the US and European heart associations have recommended changing RAS-inhibitor administrations. The BRACE CORONA and REPLACE COVID randomized trials contributed to support the theory that the need for the medications was a marker for patients who have a greater underlying burden of chronic health conditions and cardio-vascular risk rather than the fact that the medications themselves were probably not causing any harm [34,35]. However, the REMAP-CAP study has found that the administration of RAS inhibitors could attenuate Ang II levels and thus improve outcomes.

If ACE2 is bound to SARS-CoV-2, the normal conversion of Ang II to Ang (1–7) would be inhibited, and there would be a relative increase in Ang II activity. The addition of TXA-127, a synthetic Ang (1–7), to COVID therapy in the hope of achieving RAS balance, would limit the effects of Ang II. Another solution is TRV-027, an agonist of the Ang II type 1 receptor [34,35]. However, these two drugs have not worked as expected. The REMAP-CAP results provide conclusive proof that this approach is not appropriate for acute treatment [31]. Despite this, there is still a possibility that RAS inhibitors could be used in a randomized trial to treat long COVID, whereas RAS dysregulation has been considered to be a cause.

The treatment of corticosteroids has no impact on persistent symptoms in hospitalized patients because long COVID-19 displays multiorgan symptoms that are likely brought on by a variety of mechanisms [36].

## 4. RAS Involvement in COVID Cardiovascular Injuries

In the cardiovascular system, RAS is involved in the pathogenesis of COVID-19 through the following pathways:

### 4.1. Inflammatory Response

Infection with the SARS-CoV-2 virus has introduced a new paradigm in the understanding of the immune response through the extent and diversity of the body disorders. Considered from a broad point of view, cellular infection with SARS-CoV-2 causes excessive production of virions and secondary endoplasmic reticulum stress [37]. To interrupt the massive production of virions, the infected cell dies as part of the defense mechanism [38]. ACE2 plays a role in mediating the interaction between the virus and host cells, being the entry receptor for the SARS-CoV-2 virus [39]. From a physiological perspective, ACE2 regulates the RAS, which plays a role in the homeostasis of the human body. ACE2 expression is more pronounced in alveolar epithelial cells, renal tubular cells, Leydig cells as well as in cells in the gastrointestinal tract or heart [40]. Furthermore, cardiomyocytes and type II alveolar cells have the ability to positively influence the transmission and generation of viruses thus increasing the vulnerability of both organs [41]. It should be noted that the effects of RAS, whether determined by local or systemic components, are hormonally influenced, and thus there is a gender-related variability.

Following the pathogenic processes, after infection, viral replication, and migration, multiple inflammatory pathways are triggered, through which macrophages and dendritic cells are activated and proinflammatory cytokines and chemokines are secreted; there are multiple inflammatory mechanisms that induce deregulation of the RAS. These processes are often excessive and self-induced and can be responsible for tissue destruction and host death, as follows:

4.1.1. Secretion of inflammatory factors by macrophages is mediated by SARS-CoV-2 S, N, and E proteins [42]. Protein N is responsible for enhancing the secretion of inflammatory factors such as TNF-alpha, interleukin (IL) 6, and IL-10 [43]. It has been suggested that the S protein may bind to Toll-like receptor (TLR) 4, which in turn increases ACE2 expression, and the S1 subunit induces activation of nuclear factor-κB (NF-*κ*b) and mitogen-activated protein kinase (MAPK) pathways [42,44].

4.1.2. Binding of Ang II to target cells activates the Janus kinase 2/signal transducer and activator of transcription 3 (JAK2/STAT) (STAT1/2/3) pathway, resulting in increased production of proinflammatory cytokines [45]. Activation of this pathogenic pathway leads to the loss of the ACE2 receptor and RAS dysfunction [46].

4.1.3. Through the p38 MAPK pathway, the immune response and ACE2 endocytosis are impaired, leading to ACE2 down-regulation, RAS disruption, and Ang II accumulation [47]. As an exacerbation of inflammatory processes occurs, the extracellular domain of ACE2 breaks down, altering RAS balance, which ultimately induces myocarditis, cardiovascular complications, and ARDS [48]. Ang II plays a role in increasing cardiomyocyte contractility and the transformation of fibroblasts in the heart into myofibroblasts with pro-fibrotic action [49]. As inflammatory processes are sustained over time and exacerbated by Ang II and oxidative stress, cardiac remodelling, and destructive effects on the vasculature occur [40].

An important factor in the pathogenesis of long COVID is oxidative stress. The main pathogenic processes of long COVID include a systemic hyperinflammatory state and coagulopathy, which are exacerbated by inflammation and oxidative stress. There is a vicious cycle of oxidative stress, inflammation, and long-COVID disease progression because thrombosis and inflammation lead to the reactivation of reactive oxygen species (ROS). Inflammation is triggered by ROS, which also affect the endothelium, cause microthrombi and neuroinflammation, stimulate the production of autoantibodies, and interfere with the synthesis of neurotransmitters [50]. Through activating NADPH Oxidase, ACE2 stimulates oxidative stress, an effect that is mainly mediated by Ang II [51]. An increase in Ang II and ROS set by the SARS-CoV-2 infection triggers oxidative stress and multisystemic injuries [52]. According to a study, the degree of severity of the disease and poor outcomes are associated with higher levels of oxidative stress markers, lower levels of antioxidant indicators, and lower levels of ACE2 expression in hospitalized COVID-19 patients [53].

Once these imbalances and fibrotic remodelling processes are initiated, RAS dysfunction and serum ACE2 activity appear to be hardly and incompletely reversible. As a result, the disturbances described by long-COVID syndrome, produced not only after severe infections but also after persistent, subclinical infections, are associated with RAS dysfunction [54].

The range of cells that can be infected by the virus is also notable, with viral genes being detected in the cardiac conduction system, which explains the arrhythmias observed in these patients [55]. A study published by Tavazzi et al. reveals, by analysis of biopsy specimens, that myocardial interstitial cells undergo viral invasion and inflammation [56]. Studies on the presence of viral particles in cells of the cardiovascular system are not entirely clear. A study that followed the morphological analysis of 276 patients who died after infection with different variants of concern of SARS-CoV-2 virus revealed the presence of spike protein, but no viral RNA has been found in the myocardium. Electron microscopy observed virion-like particles on the surface of the vascular endothelium [57].

Based on single-cell RNA sequencing and histology studies, human cardiomyocytes have been found to express the SARS-CoV-2 receptor ACE2, especially in patients with cardiovascular disorders, which suggests that SARS-CoV-2 may target cardiomyocytes [58]. In the cytoplasm and nucleus, cardiomyocytes present a low level of TMPRSS2 [59], but significant levels of cathepsin B and L, indicating that endocytosis is the primary route for SARS-CoV-2 entry by cathepsin-dependent manner [58,60]. These results indicating the role of cathepsins in cardiomyocyte infection support the consideration of cathepsin L inhibitors in anti-COVID therapy [61]. Cardiomyocytes are permissive for SARS-CoV-2 infection, according to in vitro studies on human induced pluripotent stem cells (hiPS) and isolated adult cardiomyocytes as well as in vivo animal models [58]. Thus, it has been shown that cardiomyocytes produce viral spike glycoproteins and have intracellular double-stranded viral RNAs. SARS-CoV-2 infectivity was confirmed in 3D cardiospheric tissue, and increased viral RNA concentrations have been found in supernatants from infected cardiomyocytes. The expression of the viral spike protein was effectively suppressed by neutralizing antibodies, recombinant ACE2, or inhibition of the RNA polymerase with remdesivir.

A significantly greater interaction between SARS-CoV-2 and the receptor can result from the microvascular pericytes expressing a significant amount of ACE2. As a result, endothelial dysfunction is caused by the lesion of the pericytes set by the dissemination of the viral infection [62]. The microvasculature’s lining pericytes, as well as vascular smooth muscle cells, fibroblasts, and cardiomyocytes, exhibit significant cardiac expression of ACE2 [63].

In addition to the exacerbated inflammatory response, the pathogenic pathways of which in relation to RAS have been described above, the association between long COVID syndrome and cardiovascular disorders can be linked to the viral genes being incorporated into the deoxyribonucleic acid of infected cells, which results in the maintenance of procoagulant processes and other cardiovascular complications [64]. From these perspectives, at least long-term RAS imbalance and cellular remodelling are a continuous process, and the final stage of evolution is fibrosis of endothelial structures, cardiomyocytes, or the conduction system.

In addition to inflammatory mechanisms, myocardial damage may also occur as a result of vascular endothelial disorders, cardiomyocyte apoptosis, increased mechanical stress, thrombus formation, or inadequate perfusion [14].

### 4.2. Endothelial Dysfunction

A dynamic connection between the circulating blood and different tissues is achieved by the vascular endothelium. The regulation of vascular tone and maintenance of vascular homeostasis depend entirely on the vascular endothelium, an active paracrine, endocrine, and autocrine organ [65].

As a multisystem disease, COVID-19 is partly caused by damage to the vascular endothelium. After infection, there may be residual repercussions and long-term sequelae, which may be caused by chronic endothelial dysfunction [66]. These COVID-19 long-term effects have been considered the next upcoming public health global emergency, and scientific data on the size and magnitude of the situation are urgently needed to assist in the planning of a proper healthcare response. The ACE2 receptors expressed by endothelial cells are used by SARS-CoV-2 to infect the host and cause clear and distinct systemic endotheliitis [67].

Endotheliitis and infection-mediated endothelial injury can cause excessive thrombin production, inhibit fibrinolysis, and activate complement pathways, which may result in microvascular dysfunction and the deposition of micro thrombi [68].

As mentioned above, SARS-CoV-2 enters in cells via the SARS-CoV ACE2 receptor, primes the S protein via the serine protease TMPRSS2, and thus, directly infects the endothelium. In addition, an authorized TMPRSS2 inhibitor limits this entry and could be used as a therapy option [23].

Endothelial damage may be a major contributing factor to the persistence of long-COVID symptoms, together with platelet pathology and the presence of micro clots in the circulation [69].

A study has revealed higher autoantibody levels in long-COVID patients [70]. These antibodies approach antiphospholipid antibodies, which are the main cause of antiphospholipid syndrome in the general population. The autoantibodies attach to cell surfaces, where they stimulate neutrophils, platelets, and endothelial cells, leading to thrombosis at the blood vessel wall barrier [71]. There are individuals with a history of SARS-CoV-2 infection, which have anti-ACE2 antibodies. These patients have reduced plasma levels of soluble ACE2. Additionally, exogenous ACE2 activity is inhibited by plasma from these patients. Thus, following SARS-CoV-2 infection, the ACE2 antibodies are produced and reduce ACE2 activity. This might result in more Ang II circulating, which promotes the proinflammatory status that underlies the long-COVID symptoms. Post-Acute Sequelae after SARS-CoV-2 infection may be treated with recombinant soluble ACE2 protein [70].

Endotheliitis present in COVID-19 patients may be the cause of the systemic impairment of microcirculatory function in various arterial beds and its clinical consequences. This statement supports the use of ACE inhibitors, anti-cytokine, anti-inflammatory, and statin drugs, to stop viral replication [67].

### 4.3. Microvascular Abnormalities: Hypercoagulability and Thrombus Formation

Patients with COVID-19 present infection-mediated endothelial injury, marked by increased von Willebrand factor levels and endotheliitis, characterized by the presence of activated neutrophils and macrophages in a number of vascular beds, including the lungs, kidney, heart, small intestine, and liver. These changes can trigger excessive thrombin production, inhibit fibrinolysis, and activate complement pathways, initiating thrombo-inflammation and ultimately leading to micro thrombus deposition and microvascular dysfunction.

The current literature reveals that SARS-CoV-2 infection increases a patient’s risk for thromboembolic diseases. A recent study showed that platelet hyperactivity is associated with detectable virus RNA in blood and that COVID-19 patients display higher platelet activation in vivo, as shown by enhanced integrin αIIbβ3 activation and P-selectin expression. In addition, platelets had high levels of ACE2 and TMPRSS2 expression, two essential cellular elements involved in SARS-CoV-2 cell entry, and SARS-CoV-2 and its spike protein induce platelet activity and thrombus formation via the MAPK pathway, which is downstream of ACE2. These findings imply that treatment with anti-spike monoclonal antibody and recombinant human ACE2 protein can prevent platelet activation and thrombus formation caused by SARS-CoV-2 [72].

The occurrence of microvascular dysfunction in long-COVID patients may be due to peripheral activation of ACE2 receptors or exacerbation of proinflammatory cytokines that may remain in circulation even after the infection subside [73]. ACE2 causes endothelial injury that results in endothelial dysfunction, microvascular inflammation, and thrombosis, as mechanisms of microvascular abnormalities in COVID-19 [74]. This observational study revealed that the underlying cause of angina-like chronic chest pain in people who have recovered from COVID-19 is coronary microvascular ischemia [75].

### 4.4. Fibrosis

Fibrosis is the outcome of almost all chronic inflammatory diseases. Fibrosis can be considered a consequence of a disordered wound-healing process and can be directly related to the severity of the acute event [76]. Different mechanisms of lung injury have been described in COVID-19, with both viral and immune-mediated mechanisms being involved [77]. Studies have shown that SARS-CoV-2 infection can lead to damage to epithelial structures and is associated with the presence of fibrotic tissue through excess collagen (fibrosis). Among the multiple effects of RAS that include the regulation of cell growth, apoptosis, and inflammation, tissue fibrosis is controlled. The practical existence of a tissue RAS that may be independent of the circulating RAS causes confusion in the overall interpretation of the role of this system, given the existence of alternative pathways related to active peptides or Ang II generation [15].

Given the short time since the outbreak of the COVID-19 pandemic and with efforts largely focused on acute case management, there are insufficient data to assess the long-term impact of infection, spontaneous reversibility, or potential benefits of initiating antifibrotic therapy in patients with post-infection sequelae.

RAS does not act by itself but is closely related to other inflammatory mechanisms, oxidative stress, and endothelial dysfunction, which contribute to the development and maintenance of cardiac lesions. Some effects of RAS such as synaptic remodelling, improved cell survival, cellular signal transmission, and antioxidant effects should not be omitted [78].

Fibrosis as a fibro-proliferative disease also recognizes a genetic predisposition and is correlated with chronic inflammation. In relation to post-COVID fibrosis, the transforming growth factor-β signalling pathway, the Wingless/int signalling pathway, and the Yes-associated protein/transcriptional coactivator PDZ-binding motif signalling pathway have been proposed as mechanisms of production [79]. Another pathway of fibrotic disease production is based on the release of fibroblast growth factors, transforming the growth factor released from megakaryocytes [80]. Due to intense inflammatory processes, there is a continuous release of these mediators, which can lead to inadequate remodelling and fibrosis [81], although current data suggest the influence of the thrombotic process as a precursor of pulmonary and hepatic fibrosis [82]. There are still no possibilities of therapeutic intervention due to the difficulty of separation from physiological healing processes.

Thille et al. described in a cohort of 159 autopsies of patients with ARDS that 4% of patients with a disease duration of less than 1 week, 24% of patients with a disease duration between 1 and 3 weeks, and 61% of patients with a disease duration greater than 3 weeks, developed fibrosis [83]. The repair process involves regeneration by native stem cells and deposition of connective tissue to replace areas of defect [84]. Alveolar macrophages play a central role in this process by phagocytosing alveolar debris and producing cytokines and growth factors involved in the repair pathway [85].

In SARS-CoV-2 infection, the main cause of aggravation of the disease course is considered to be cytokine storm with excessive release of metalloproteinases producing epithelial and endothelial damage, vascular endothelial cell growth factor (VEGF), and cytokines such as IL-6 and TNFα involved in the fibrotic process [86]. VEGF and fibroblast growth factors stimulate the migration and proliferation of intact endothelial cells leading to pulmonary capillary angiogenesis [87]. The detection of fibrotic changes at the beginning of the disease suggests an attempt to repair the lesions that appeared in the acute phase. However, it is too early in the disease progression to determine whether this finding resolves in time or results in permanent fibrosis.

## 5. Conclusions

We have attempted to summarize the pathophysiological mechanisms resulting from an unbalanced RAS system that may contribute to long COVID’s symptoms.

However, further research on the underlying mechanisms is needed. Recent data suggest an intricate immune-inflammatory signaling network involving the imbalance of the RAS. To date, there is not yet a biomarker for the long-COVID prediction. Due to the wide range of symptoms, it is important to thoroughly evaluate the patients in order to exclude any other causes or comorbidities, notably persistent inflammation, microvascular cardiac dysfunction, microclots, persistent endothelial dysfunction, and fibrosis, which have been all described in long COVID.

The variety of cardiovascular symptoms in long COVID requires extensive evaluation and assessment, which challenges clinicians and determines the design of future clinical trials. Applying a variety of approaches to reset the RAS may be a possibility for managing patients with long COVID.

Association between long-COVID syndrome and cardiovascular disorders can be linked to the viral genes being incorporated into the deoxyribonucleic acid of infected cells, which results in the maintenance of a vicious cycle of oxidative stress, inflammation, as well as procoagulant processes. Microvascular dysfunction in long-COVID patients may be due to peripheral activation of ACE2 receptors or exacerbation of proinflammatory cytokines that may remain in circulation even after the infection subsides. Long-term RAS imbalance and cellular remodelling is a continuous process, and the final stage of evolution is fibrosis of endothelial structures, cardiomyocytes, or the conduction system. Therapeutical approaches that rebalance the RAS may be able to reduce the hyperinflammatory and procoagulant state experienced by long-COVID patients.

Long COVID continues to concern the medical world and compels us to expand the diagnostic methods and therapeutic guidelines to protect patients from the harmful effects of SARS-CoV-2 proteins. Multi-organ damage and cardiovascular complications in particular require detailed genetic and molecular studies, as well as translational and randomized trials.

## 6. Future Perspectives

Long-term longitudinal follow-up of cardiovascular dysfunction in patients with long COVID is needed to better understand the mechanism behind the persistence of symptoms and to target therapy.

The connection between endothelial dysfunction and long-COVID-19 symptoms is becoming clearer. The medical care of long COVID patients may benefit from dividing risks into categories.

Because of the particular nature of long-COVID cardiovascular symptoms, it is likely that several methods of therapy will be necessary to manage care for the patients.

Studies on the biological factors that could lead to the onset of long-COVID symptoms are required. These factors may include the presence of SARS-CoV-2 persistent reservoirs in specific tissues, the reactivation of other pathogens under immune dysregulation, the interaction of viruses with hosts, the host microbiome with the human virome, coagulation disorders, and autoimmunity triggered by peptides from the pathogen and the host that are structurally similar.

Thus, teams with a variety of expertise, such as pathology, virology, immunometabolism, physical therapy, and rehabilitation, must collaborate on studies on long-COVID patients.

## Data Availability

Additional data are available from the corresponding author upon reasonable request.
